# *Stipa tenacissima* L.: A New Promising Source of Bioactive Compounds with Antioxidant and Anticancer Potentials

**DOI:** 10.3390/life11080757

**Published:** 2021-07-27

**Authors:** Mehdi El Bouchti, Mohammed Bourhia, Amal Alotaibi, Kaoutar Aghmih, Sanaa Majid, Riaz Ullah, Ahmad Mohammad Salamatullah, Mounir El Achaby, Mina Oumam, Hassan Hannache, Omar Cherkaoui, Mohammed El Mzibri, Laila Benbacer, Said Gmouh

**Affiliations:** 1 Laboratory REMTEX, Higher School of Textile and Clothing Industries, km 8, Route d’El Jadida, Oulfa, Casablanca B.P. 7731, Morocco; melbouchti@gmail.com (M.E.B.); ag.kaoutar@gmail.com (K.A.); cherkaoui@esith.ac.ma (O.C.); 2 Laboratory of Chemistry-Biochemistry, Environment, Nutrition, and Health, Faculty of Medicine and Pharmacy, Hassan II University, Casablanca B.P. 5696, Morocco; 3 Basic Science Department, College of Medicine, Princess Nourah Bint Abdulrahman University, Riyadh 11564, Saudi Arabia; 4 Laboratory of Materials Engineering for Environment and Valorization (GeMEV), Aïn Chock Faculty of Sciences, Hassan II University, Casablanca B.P. 7731, Morocco; sanaamajid2013@gmail.com; 5 Department of Pharmacognosy (MAPPRC), College of Pharmacy, King Saud University, Riyadh 11564, Saudi Arabia; rullah@ksu.edu.sa; 6 Department of Food Science & Nutrition, College of Food and Agricultural Sciences, King Saud University, P.O. Box 2460, Riyadh 11451, Saudi Arabia; asalamh@ksu.edu.sa; 7 Materials Science and Nano-Engineering Department, Mohammed VI Polytechnic University, Lot 660–Hay Moulay Rachid, Ben Guerir 43150, Morocco; mounir.elachaby@um6p.ma; 8 Laboratory of Engineering and Materials LIMAT, Faculty of science Ben M’Sik, Hassan II University, Casablanca 7955, Morocco; oumam.uh2c@gmail.com (M.O.); hannache.hassan@gmail.com (H.H.); said.gmouh@gmail.com (S.G.); 9 Biology and Molecular Research Unit, Department of Life Sciences (CNESTEN), Rabat B.P. 10001, Morocco; mzibri@yahoo.com (M.E.M.); Benbacer@cnsten.org.ma (L.B.)

**Keywords:** *Stipa tenacissima* L., methanol extract, bioactive compounds, antiproliferative activity, antioxidant properties

## Abstract

Background: *Stipa tenacissima* L. (*S. tenacissima*), called Esparto grass, is a cultivated species used for industrial purposes, including textile production. This species has never been studied for its medical potential before, nor has it been used in traditional medicines. It is thus fitting that the present study aimed to investigate the pharmacological potential of *S. tenacissima.* To achieve this goal, this work was conducted to study the chemical composition, antioxidant properties, and antiproliferative effects of *S. tenacissima* against cancerous cell lines, including the human colorectal adenocarcinoma cell line (HT-29) and human breast adenocarcinoma cell line (MDA-MB-231). Fractionation and characterization of *S. tenacissima* extract showed the presence of promising bioactive fractions. The fractions obtained from *S. tenacissima* extract exhibited interesting antioxidant properties, with IC_50_ values ranging from 1.26 to 1.85 mg/mL. All fractions, such as F1, F2, F3, and F4, induced an important antiproliferative effect on the cancer cell lines MDA-MB-231, scoring IC_50_ values ranging from 63.58 ± 3.14 to 99.880 ± 0.061 µg/mL. These fractions (F1, F2, F3, and F4) also exhibited a potent antiproliferative effect versus HT-29 cell lines, with IC_50_ values ranging from 71.50 ± 4.97 to 87.500 ± 1.799 µg/mL. Therefore, *S. tenacissima* could constitute a new natural source of bioactive compounds that can be used for therapeutic purposes to fight cancer and free radical damage.

## 1. Introduction

Since prehistoric times, people have used plants for food, condiments, and therapeutic purposes. More than half of medicines placed on the market come from natural products or their derivatives [1].

The use of herbal medicine is very old, and several historical studies indicate early usage of plants for medical purposes [2]. Medicinal herbs are an inexhaustible source of natural bioactive compounds that could constitute precious tools for basic research in the treatment of various diseases. Many medicinal plants have been described for their effectiveness in curing several diseases, such as hypertension, degenerative diseases, diabetes, viral infections, and cancer [3,4,5]. Natural medicines could be used in the decoction format of the whole plant, extracts of special plant parties, or purified derivatives. The aim is no longer the same between the know-how of old civilizations and today’s technology. Nowadays, purifications using chromatographic techniques allow the identification and isolation of active compounds, and eventually a synthetic production of new bioactive natural substances [6].

*Stipa. tenacissima* L. is a typically Mediterranean perennial herb that grows in North Africa, especially in the highlands of Morocco and Algeria. It is a xerophilic plant that constitutes one of the most representative vegetation types in the dry Mediterranean ecosystems. It represents the most available source of fiber production in the region [1]. It constitutes a natural barrier against desertification and desert encroachment in the Maghreb region, and, therefore, plays an important role in ecosystem protection [2].

In North African countries, *Stipa tenacissima* L. is called Esparto grass, which is widely used as a potential reinforcement of polymer matrices. Furthermore, it is mainly used in the high-quality paper industry as a noble raw material due to its specific properties, since reinforcements in composite materials require a powerful adhesion between the synthetic matrix and the fiber [3]. It increases the resistance to moisture [4], and it is used for the production of cellulose nanofibers [5]. *Stipa tenacissima* L. is very rich in cellulose, hemicellulose, and lignin [6]. It also contains small amounts of proteins and inorganic compounds [7]. Some unsaturated fatty acids, particularly oleic and linoleic acids, are widely used in the dietetic field [8].

To the best of our knowledge, *Stipa tenacissima* L. is used for textile and paper production only [9]. Nevertheless, no data about its possible application in medicine have been reported to date. With the goal of contributing to better knowledge about *Stipa tenacissima* L. as a new promoting source of pharmacological and biological activities, the current study was the first work that aims to investigate the phytochemical composition and antioxidant and antiproliferative properties of Esparto grass aerial parts.

## 2. Materials and Methods

### 2.1. Collection of Plant Material

*Stipa tenacissima* L. was collected in July 2017 from the local region and was identified by plant taxonomist before being studied. Aerial parts were dried at room temperature before being ground into a fine powder [10].

### 2.2. Extraction and Isolation of Bioactive Compound Classes

**Figure 1:**
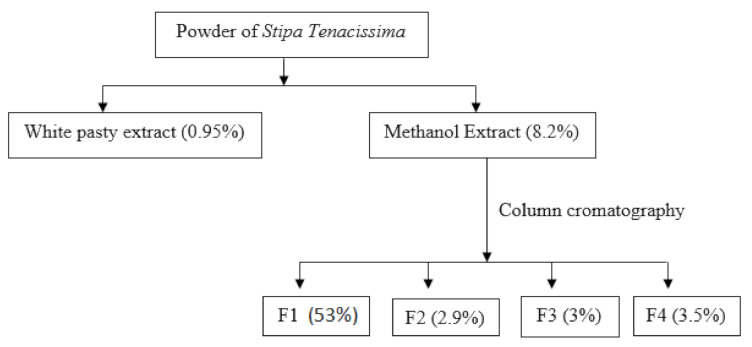
Scheme of *Stipa tenacissima* L. fractionation.

Two hundred grams of the dry plant were macerated with 3 L of methanol under reflux for 4 h. Afterward, the mixture was filtered and concentrated under reduced pressure. The organic extract (40 g) was fractionated by using column chromatography with silica gel (Kieselgel 60, 70–230 mesh ASTM). A mixture of hexane/ethyl acetate was used as eluent with different compositions (30/70, 20/80, 10/90, 0/100) of increasing polarities to get four fractions: F1, F2, F3, and F4 (Figure 1).

### 2.3. Determination of Total Phenol Contents

In the present work, the total phenolic content of the extracted fractions was studied using the Folin–Ciocalteu method [11]. Briefly, 0.1 mL of each fraction was mixed with 2 mL of 10 % diluted Folin reagent before one milliliter of 7.5% Na_2_CO_3_ solution was added. Next, the mixture was vigorously stirred and then kept for 30 min at 45 °C in the darkness. The absorbance reading was carried out at 765 nm using a vis-NIR spectrophotometer. The standard curve of gallic acid was performed with different concentrations (0.5–15 mg/L).

### 2.4. DPPH Radical Inhibition Test

The radical scavenging effect of the isolated fractions was investigated using the DPPH method [12]. Each fraction was tested with concentrations ranging from 0.5 to 5.5 mg/mL. Briefly, 0.2 mL of the sample test was added to 2.8 mL of DPPH solution (24 mg/L). After 30 min of incubation at 25 °C in the darkness, the absorbance (A) was read at 515 nm. Ascorbic acid was used as a standard reference. The inhibition of free radical DPPH was given as a percentage according to the following formula [13,14,15,16]:

% Inhibition= (A_C_ − A_D_)/Ac × 100

where A_C_ is the absorbance of the blank control and A_D_ is the absorbance of each fraction at different concentrations. The IC_50_ value (the concentration required to inhibit 50% of DPPH) was calculated from the dose response curve [17,18,19].

### 2.5. Cancer Cell Lines and Culture Conditions

The potential antiproliferative effect of *stipa tenacissima* L. fractions was tested on two cancerous cell lines, including human colorectal adenocarcinoma (HT-29) and human breast cancer cell lines (MDA-MB-231). Cancer cell lines were cultivated in Dubelco’s Modified Eagle’s Medium (DMEM) with 10 % (*v*/*v*) of FBS (heat-fetal bovine serum, PAA Laboratories, Austria), and 1% penicillin/streptomycin (10,000 IU/mL). Afterward, cells were cultured at 37 °C, 95% humidity, 5% carbon dioxide, and 80% confluency [20]. In this work, mitomycin was used as a drug reference for comparison purposes.

#### MTT Assay

The antiproliferative effect of different fractions of *Stipa tenacissima* L. extract on tumor cell lines was determined by the MTT colorimetric assay. In summary, when the culture reached exponential growth at a cellular density of 8000 cells/well, concentrations of each fraction ranging from 15.6 to 500.0 µg/mL were added to this culture. Cells were incubated for 48 h at 37 °C. At the end of the experimental period, 10 µL of MTT with 5 mg/mL was added to multiwall plates. Next, these plates were incubated again for an additional 4 h at 37 °C. Afterward, the reduced MTT was read at 570 nm using a microplate reader. Experiments were carried out in duplicate assays [21].

The percentage of antiproliferative effect (cell death) was calculated using the following Equation:

(1) $$\mathrm{Cell}\;\mathrm{death}\;\left(\%\right)=\frac{\mathrm{control}\;\mathrm{OD}\;-\;\mathrm{sample}\;\mathrm{OD}}{\mathrm{control}\;\mathrm{OD}}\;\times \;100$$

where control OD is the absorbance of the non-treated cells and sample OD is the absorbance of the treated cells.

### 2.6. Gas Chromatography–Mass Spectrometry (GC-MS)

*Stipa tenacissima L.* extract fractions were characterized using GC-MS. A Claus 580 chromatography apparatus with a capillary column of 5% phenyl and 95% methyplisyloxane (30.0 m × 250 μm), coupled with a mass spectrometer (MS), was used in this assay. Helium gas was involved as a carrier. The split was chosen to 1/75, while the injection volume was 1 µL. In this sense, the injection and detection temperatures were, respectively, set to 250 and 280 °C. Moreover, the temperature of the regulating furnace was programmed as follows: from 50 °C to 200 °C with a rate of 11 °C/min and then from 200 °C to 240 °C with a rate of 6 °C/min. The identification was conducted by comparing the spectrum along with the retention time of compounds with those of the NIST library [22].

### 2.7. Statistical Analysis

The present data were expressed as the means of triplicate assays ± SD (standard deviation). The significance values of the difference between the averages were deemed using ANOVA and GraphPad Prism 7 software. The averages of the investigated data were compared using the Holm Sidak Test. Statistically, a significant difference was considered at *p* < 0.05.

## 3. Results and Discussion

### 3.1. Extraction Yield and Fractionation of Natural Organic Compounds from Stipa tenacissima L. Extract

The yield of the extract obtained from *Stipa tenacissima* L. after the maceration with methanol was 8.2%. Fractionation of the organic extract of *Stipa tenacissima* L. produced four fractions: F1, F2, F3, and F4, with yields of 53%, 2.9%, 3%, and 3.5%, respectively. Analysis of variance used for statistical purposes showed no significant difference between fractions F2, F3, and F4 concerning the extraction yield (*p* > 0.05). However, a significant difference was observed between F1 and F2, F3, and F4 (*p* *** < 0.05).

### 3.2. Determination of Total Phenolic Compounds

The results of the total phenolic compounds contained in the isolated fractions F1, F2, F3, and F4 are summarized in Table 1, where the total phenolic content is expressed in gallic acid (GA) equivalent per 100 g of dry extract. The phytochemical analysis revealed the presence of phenolic compounds in the four evaluated fractions. As reported in Table 1, F3 and F4 fractions showed high phenolic content (31.790 ± 0.008 and 31.2600 ± 0.0005 g GA/100 g, respectively) when compared to F1 and F2 fractions (5.530 ± 0.003 and 18.120 ± 0.005 g GA/100 g, respectively). Regarding the total polyphenolic content, the statistical analysis showed no significant difference between fractions F3 and F4 (*p* > 0.05). However, a significant difference was observed between fraction F1 and fractions F2, F3, and F4 (*p* ** < 0.05).

The phenolic compounds are the major active classes responsible for antioxidant properties [13]. In the last few decades, research for natural antioxidant substances has become interesting, owing to their role in the protection of health. In this sense, polyphenols have been widely reported to have great antioxidant potential, along with radical scavenging ability [23].

### 3.3. Antioxidant Activity by DPPH

The antioxidant capacity of the four fractions was evaluated using the DPPH method. The IC_50_ values of DPPH scavenging activities of each fraction were compared to IC_50_ of ascorbic acid as a positive reference. Table 2 reports the DPPH radical scavenging activity results of the assessed fractions of *Stipa tenacissima* L. extract. The present fractions showed interesting antioxidant properties, with IC_50_ values ranging from 1.26 to 1.85 mg/mL. Regarding the investigated antioxidant activity, the analysis of variance showed no significant difference between fractions F2, F3, and F4 (*p* > 0.05). However, a significant difference was observed between all tested fractions F1, F2, F3, and F4 and the ascorbic acid (*p* *** < 0.05). The antioxidant properties reported in these fractions could be due to the high amount of polyphenols determined by the chemical analysis, which were consistent with the results reported in the literature [24,25].

**Figure 2:**
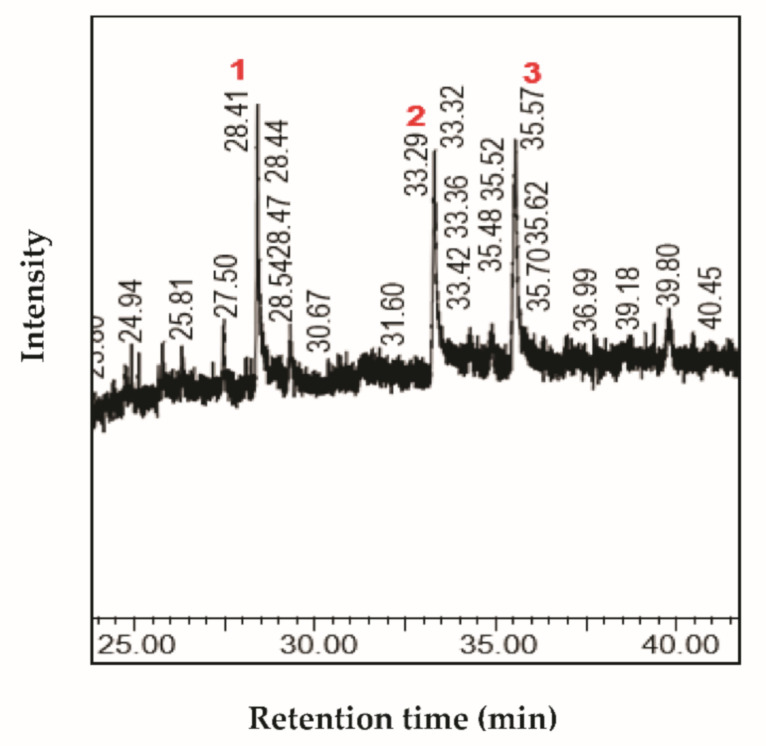
Chromatogram profile of compounds identified in the fraction F1 from *Stipa tenacissima* L.

**Figure 3:**
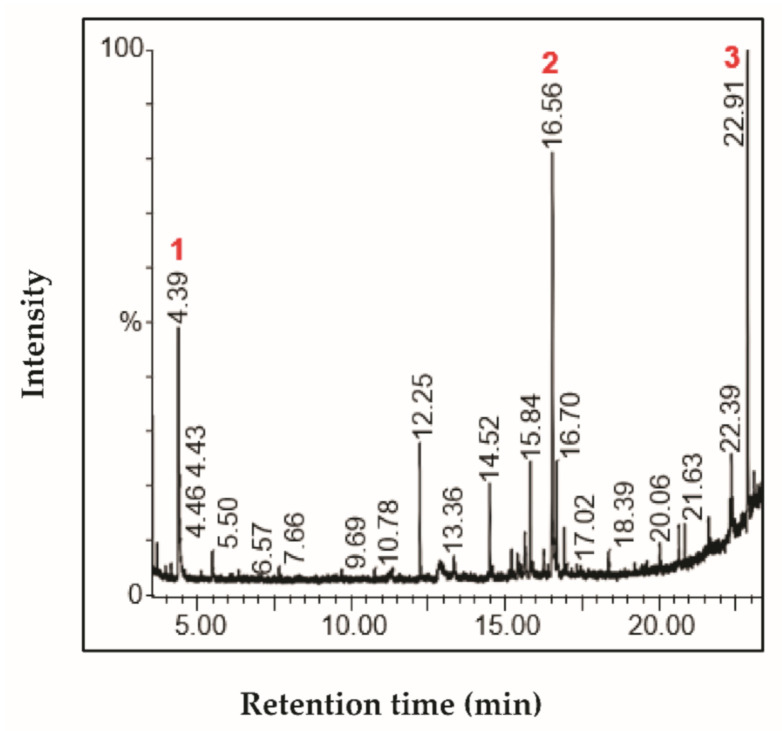
Chromatogram profile of compounds identified in the fraction F2 from *Stipa tenacissima* L.

**Figure 4:**
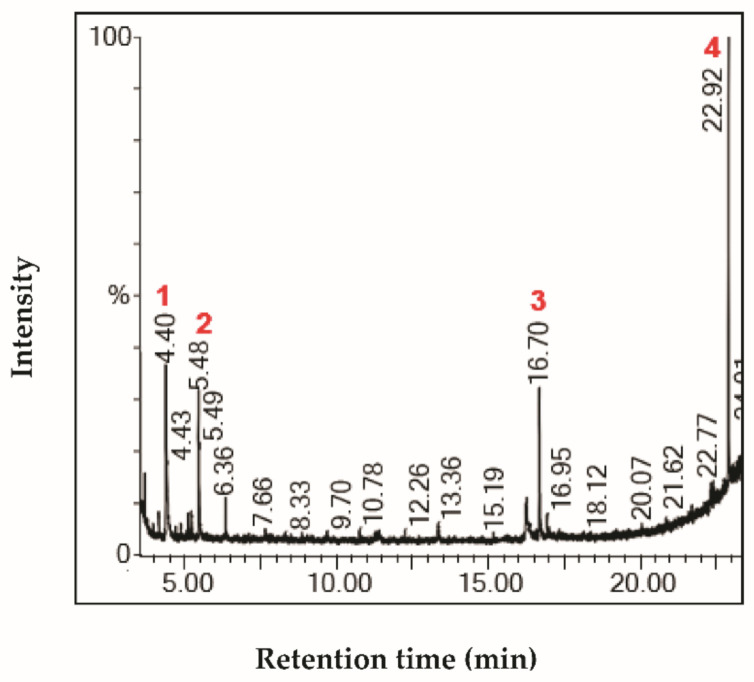
Chromatogram profile of compounds identified in the fraction F3 from *Stipa tenacissima* L.

**Figure 5:**
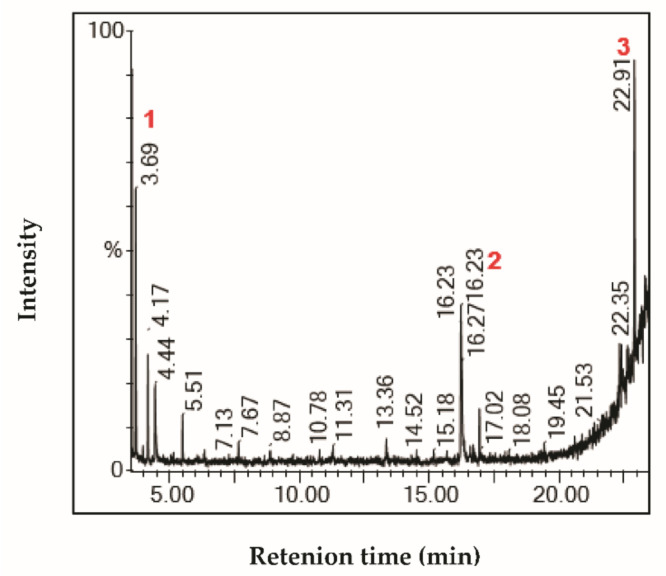
Chromatogram profile of compounds identified in the fraction F4 from *Stipa tenacissima* L.

The characterization of fractions by GC-MS showed the presence of interesting bioactive compounds contained in each fraction. The analyzed fractions showed different bioactive constituents, which differed according to the extraction solvent. The major identified compounds in each fraction (F1, F2, F3, and F4) are presented in Table 3, Table 4, Table 5 and Table 6 and Figure 2, Figure 3, Figure 4 and Figure 5.

### 3.4. GC-MS Analysis

The GC-MS analysis of fraction F1 from *Stipa tenacissima* L. extract revealed the presence of four major compounds, including γ-sitosterol and lupeol (Table 3; Figure 2). It was reported that γ-sitosterol and lupeol are biologically active. γ-Sitosterol belongs to the plant sterols, which were previously discovered in *Girardinia heterophylla* extracts [26]**.** γ-Sitosterol can reduce hyperglycemia and inhibits glycogenesis [27]**.** The anticancer properties of γ-sitosterol and beta-sitosterol (an epimer of γ-sitosterol) against various cancerous cell lines were also reported elsewhere [28]**.** Plant species that contain sitosterols are recommended for cancer treatment [29]**.** γ-Sitosterol induced an antiproliferative effect on cancerous cells through the downregulation of c-myc expression, along with the induction of the apoptotic pathways [30]. Another major compound detected by GC-MS in the F1 fraction was lupeol, a phytosterol widely common in medicinal plants. The latter currently arouses great interest due to its biological and therapeutic potential. Lupeol possesses anti-inflammatory and anti-tumor activities, as reported in a study published by Hata et al. [24].

The main compounds identified in the fraction F2 were diacetone alcohol, loliolide, octadecane, and 3-ethyl-5-(2-ethylbutyl) (Table 4; Figure 3). Loliolide, a monoterpenoid hydroxylation, exhibits anticancer, antifungal, antibacterial, and antioxidant properties as reported elsewhere [25]**.** It is also used to treat dysentery and diarrhea [26].

The major compounds identified in F3 fraction were diacetone alcohol, pentoxone, 5,5,8a-trimethyl-3,5,6,7,8,8a-hexahydro-2H-chromen, and bis(2-propylpentyl)benzene-1,2-dicarboxylate (Table 5; Figure 4). It has been reported in the literature that diacetone alcohol has pharmacological activity, including antiproliferative properties [29]**.**

GC-MS analysis of fraction F4 revealed the presence of three major compounds, including methyl sulfolane, coniferyl alcohol, and 2-(benzyloxy)-3, 6-difluorophenol (Table 6; Figure 5). Presently, no additional data have been reported on these compounds in terms of pharmacological activities.

### 3.5. Antiproliferative Activity of Fractions of Stipa tenacissima L. Extract on Cancer Cells

Despite some advances in cancer treatments, some types of cancer resist several synthesized drugs. In this sense, it was reported that natural drugs derived from plants can be effective against cancer. Even though several natural anti-cancer compounds have been tested, plants are still a non-limited source of bioactive compounds. Thus, a large unexplored plant species deserves to be studied in terms of medicinal potential [14].

**Figure 6:**
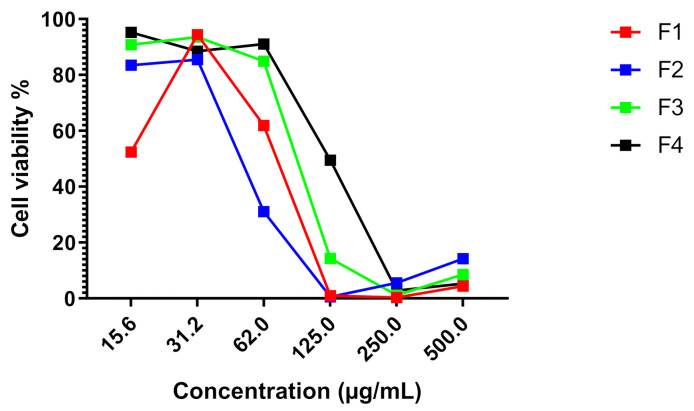
Effect of *Stipa tenacissima* L. extract fractions on HT-29 cell proliferation at 72 h post-treatment.

**Figure 7:**
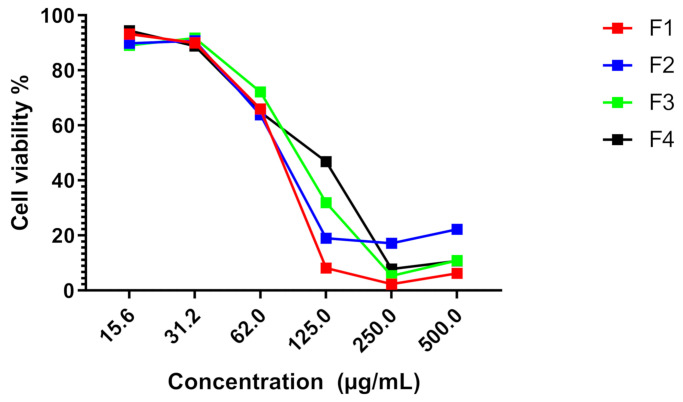
Effect of *Stipa tenacissima* L. extract fractions on MDA-MB-231 cell proliferation at 72 h post-treatment.

**Figure 8:**
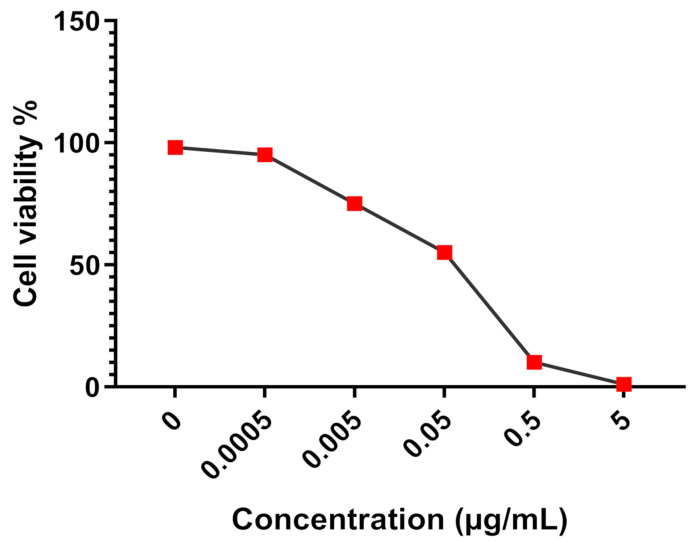
Antiproplifertaive effect of mitomycin on cancer cell lines.

**Figure 9:**
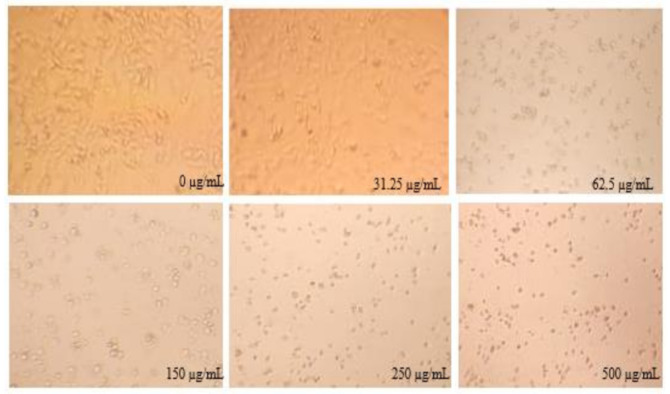
Appearance of cancer cell mortality after being treated with different concentrations of *Stipa tenacissima* L. fractions.

*Stipa tenacissima* L. is a cultivated species used in textile production. It has never been investigated in terms of medicinal potential. Therefore, the current research work was the first report on the antiproliferative effect of *Stipa tenacissima* L. extract fractions. Using cell viability indices, the MTT test revealed that the four fractions reduced cell viability in both cancer cell lines in a dose-dependent manner at 72 h post-treatment (Figure 6, Figure 7, Figure 8 and Figure 9).

Fractions F1, F2, F3, and F4 induced an important antiproliferative effect against both cancer cell lines MDA-MB-231 and HT-29 HT. The results are summarized in Table 7.

Regarding the IC_50_ values of the antiproliferative effect induced by fractions F1 and F2 on both MDA-MB-231 and HT-29 cell lines, the statistical analysis showed a significant difference between the IC_50_ values of fractions F1 and F2 towards both MDA-MB-231 and HT-29 when compared to F3 and F4 (*p* * < 0.05). The mitomycin used as a drug reference for comparison purposes showed a strong antiproliferative effect towards all cancer cell lines tested with IC_50_, scoring 0.05 µg/mL (Figure 8).

The GC-MS analysis of the F1 fraction showed the presence of sterols, such as γ-sitosterol and lupeol. These compounds could be responsible for the antiproliferative effects exhibited by the fraction. The present findings were in agreement with those reported in earlier work, which showed that γ-sitosterol and lupeol possess potential cytotoxic effects [27,28]. The antiproliferative effect of the F2 fraction may be due to loliolide and diacetone alcohol, which might have antiproliferative effects [18]. Regarding the bioactive compound classes detected in the F3 and F4 fractions, no previous literature has reported their potential antiproliferative effects, hence, it seems that new compounds from *Stipa* *tenacissima* L. exhibit antiproliferative effects on MDA-MB-231 and HT-29 cancer cell lines.

## 4. Conclusions

The present research work provides data about newly bioactive fractions from the organic extract of *Stipa tenacissima* L., which is currently used for textile production. The plant fractions showed high antioxidant properties, along with high antiproliferative effects on human colorectal adenocarcinoma cell MDA-MB-231 and human breast adenocarcinoma cell HT-29. *Stipa tenacissima* L. could constitute a new way of conceptualizing effective drugs against cancer and free radical damage.

## Figures and Tables

**Table 1:** Total polyphenolic content contained in the separated fractions from *Stipa tenacissima* L. extract.

Fractions	g GA/100 g
F_1_	5.530 ± 0.003
F_2_	18.120 ± 0.005
F_3_	31.790 ± 0.008
F_4_	31.2600 ± 0.0005

**Table 2:** IC_50_ values of different fractions isolated from *Stipa tenacissima* L. extract.

Fractions and Antioxidant Standard	IC_50_ (mg/mL)
F_1_	N.D
F_2_	1.85
F_3_	1.26
F_4_	1.65
AA	0.04

**Table 3:** Organic compounds identified in the fraction F1 of *Stipa tenacissima* L.

Peak Number	Retention Time (min)	Compound Name	Molecular Formula	CAS Number	Peak Area (%)
1	28.42	Heneicosyl formate	C_22_H_44_O_2_	77508-65-7	2.54
2	33.31	gamma-Sitosterol	C_29_H_50_O	83-47-6	2.73
3	35.55	Lupeol	C_30_H_50_O	545-47-1	2.88

**Table 4:** Organic compounds identified in the fraction F2 of *Stipa tenacissima* L.

Peak Number	Retention Time (min)	Compound Name	Molecular Formula	CAS Number	Peak Area (%)
1	4.39	Diacetone alcohol	C_6_H_12_O_2_	123-42-2	2.17
2	16.55	Loliolide	C_11_H_16_O_3_	73410-02-3	2.24
3	28.18	Octadecane, 3-ethyl-5-(2-ethylbutyl)	C_26_H_54_	55282-12-7	0.96

**Table 5:** Organic compounds identified in the fraction F3 of *Stipa tenacissima* L. extract.

Peak Number	Retention Time (min)	Compound Name	Molecular Formula	CAS Number	Peak Area (%)
1	4.39	Diacetone alcohol	C_6_H_12_O_2_	123-42-2	1.88
2	5.47	Pentoxone	C_7_H_14_O_2_	107-70-0	0.93
3	16.70	5,5,8a-Trimethyl-3,5,6,7,8,8a-hexahydro-2H-chromen	C_12_H_20_O	54344-82-0	1.10
4	22.92	Bis (2-propylpentyl) benzene-1,2 dicarboxylate	C_24_H_38_O_4_	70910	2.45

**Table 6:** Organic compounds identified in the fraction F4 of *Stipa. tenacissima* L. extract by GC-MS.

Peak Number	Retention Time (min)	Compound Name	Molecular Formula	CAS Number	Peak Area (%)
1	3.69	Methyl sulfolane	C_5_H_10_O_2_S	1003-46-9	0.52
2	16.23	Coniferyl alcohol	C_10_H_12_O_3_	32811-40-8	0.78
3	22.35	2-(Benzyloxy)-3,6-difluorophenol	C_13_H_10_F_2_O_2_	152434-80-5	0.64

**Table 7:** IC_50_ values of *Stipa tenacissima* L. fractions versus MDA-MB-231 and HT-29 cell lines.

Stipa tenacissima L. Fractions	IC_50_ Values (µg/mL)	
	MDA-MB-231	HT-29
F1	63.58 ± 3.14	71.50 ± 4.97
F2	72.410 ± 0.015	77.70 ± 1.62
F3	93.320 ± 0.041	87.350 ± 0.031
F4	99.880 ± 0.061	87.500 ± 1.799

## Data Availability

All data reported here are available from the authors upon request.

## Abbreviations

DPPH	1,1-diphenyl-2-picryl-hydrayl
DMEM	Dulbecco’s Modified Eagle’s Medium
FBS	Fetal bovine serum
MTT	Methylthiazol tetrazolium
NIST	National Institute of Standards and Technology
GC-MS	Gas chromatography–mass spectrometry
GA	Gallic acid
IC_50_	Half-maximal inhibitory concentration
AA	Ascorbic acid
OD	Optical density
